# Comparative Proteomic Analysis of Young and Adult Bull (*Bos taurus*) Cryopreserved Semen

**DOI:** 10.3390/ani11072013

**Published:** 2021-07-05

**Authors:** Błażej Westfalewicz, Mariola Słowińska, Sylwia Judycka, Andrzej Ciereszko, Mariola A. Dietrich

**Affiliations:** Department of Gamete and Embryo Biology, Institute of Animal Reproduction and Food Research, Polish Academy of Sciences, Tuwima 10, 10-748 Olsztyn, Poland; b.westfalewicz@pan.olsztyn.pl (B.W.); m.slowinska@pan.olsztyn.pl (M.S.); s.judycka@pan.olsztyn.pl (S.J.); a.ciereszko@pan.olsztyn.pl (A.C.)

**Keywords:** *Bos taurus*, semen, cryopreservation, proteome, maturation

## Abstract

**Simple Summary:**

In this study, we compared sperm quality parameters and proteomic profile of the cryopreserved semen (spermatozoa and supernatant) obtained from young (2 years old) and adult (4 years old) Holstein Friesian bulls. We found differences in proteome composition between sperm from young and adult bulls and identified important age-related proteins both in spermatozoa and supernatant after thawing. Our results indicate higher maturity of adult bull spermatozoa compared to the spermatozoa of young bulls, which might contribute to an increased ability to fertilize an oocyte. On the other hand, young bull spermatozoa were equipped with proteins involved in cytoskeleton development, suggesting that developmental processes are still in progress.

**Abstract:**

The age of the bull is widely accepted to influence the production of sperm, affecting the amount and quality of produced semen, which in turn impacts the results of cryopreservation. However, the exact influence of the maturation process on cryopreserved sperm, as well as the underlying molecular mechanisms of this process, are not fully understood. The goal of this study was to evaluate changes in the proteome of thawed semen (spermatozoa and supernatant) collected from young and adult bulls (*n* = 6) using the 2D-DIGE approach. The quality of semen was assessed using a CASA system and flow cytometry. We found no significant age-related variation in semen quality, with the exception of the average path velocity of sperm movement, which was higher in adult bulls. Proteomic analysis indicated 15 spermatozoa proteins and 10 supernatant proteins with significant age-related changes. Our results suggest that semen from adult bulls is better equipped with proteins related to energy production, protection of spermatozoa against oxidative stress and fertilizing ability. Proteins increased in abundance in young bull spermatozoa were connected to the cytoskeleton and its development, which strongly suggests that developmental processes are still in progress. In conclusion, our results provide novel insight into the mechanism of the development of the male reproductive system of cattle.

## 1. Introduction

The breeding of cattle largely revolves around the utilization of cryopreserved semen in artificial insemination, which leads to reduced costs and greater ease of semen distribution [1]. For successful insemination, the use of consistent and high-quality cryopreserved semen is required. Variability in cryopreservation success is dependent on various factors, including the age of bulls.

The age of the bulls is widely accepted to influence the production of sperm, particularly affecting the amount and quality of produced semen [2]. Almost all available data suggests that three-year-old bulls, up to four to six years of age, produce larger quantities of higher quality semen compared to younger bulls [3,4,5,6]. Compared to older bulls, the semen of younger bulls is described by characteristics such as higher susceptibility to seasonal changes [7] or a higher percentage of spermatozoa with proximal droplets [8]. It must be emphasized that the differences in semen quality between young and adult bulls are not always consistent and can vary with respect to climate zone, breed and rearing and handling conditions [3,9,10,11]. The source of these differences is presently unknown. For this reason, research into the mechanisms related to the maturation of the reproductive tract is justified.

The proteomic approach allows for an in-depth, comprehensive identification of proteins and a comparative analysis. This unique perspective offers insight into the molecular mechanisms underlying various processes, such as the maturation of semen. Proteomic studies have already been utilized in bull reproduction research. Attempts to describe the proteome of spermatozoa [12], seminal plasma [13,14,15] and other parts of the reproductive tract, such as the seminal vesicles [15] and epididymis [16,17,18], have been made. Many works have focused on researching the fertility-related proteins in semen [12,19,20,21,22,23], as well as the connection between proteome and cryopreservation [24,25,26,27,28,29]. However, according to our best knowledge, no proteomic study has been conducted to investigate the influence of age on bull semen. The goal of this study was to characterize semen quality parameters and to evaluate changes in the proteome of cryopreserved semen (spermatozoa and supernatant after cryopreservation) collected from young (two years old) and adult (four years old) Holstein Friesian bulls (*n* = 6 bulls in each group) using two-dimensional differential in-gel electrophoresis method (2D-DIGE). Classification of bulls’ age was in agreement with Trevizan et al. [11], who defined bulls aged 1.8–2 years and 3.5–7.0 years as young and mature, respectively. Similarly, Mandal et al. [9] divided bulls into two classes that are young (up to 30 months) and adult (31 to 70 months).

## 2. Materials and Methods

### 2.1. Semen Collection

The experiment was carried out on commercially available cryopreserved semen collected from the same six Holstein Friesian bulls at the age of two (young bulls) and four (adult bulls). Semen was collected at the Animal Breeding and Insemination Centre (Olecko, Poland) as previously described [26,30]. The minimal sperm parameters for cryopreservation were as follows: sperm motility ≥ 70%, sperm concentration ≥ 1 × 10^9^ spermatozoa/mL and a quality of mass movement of spermatozoa graded as at least 2 on a 3-point scale. Before cryopreservation, semen was extended with BIOXcell extender (IMV Technologies, L’aigle, France) [26] to a final concentration of 12 × 10^6^ spermatozoa/250 μL straws and equilibrated at 4 °C for 2.5 h. Then, straws were frozen using Digitcool 5300 freezer (IMV Technologies), according to the freezing curve (Supplementary Figure S1). Straws were thawed at 37 °C for 1 min.

### 2.2. Extraction of Proteins from Bull Semen

An aliquot of 0.5 mL thawed semen from each bull was subjected to centrifugation as described previously [26]. The supernatant of cryopreserved semen contains seminal plasma proteins, protein-free extender and substances released from spermatozoa after freezing/thawing. In our previous work the BIOXcell extender did not show any proteins visible in the gel [26]. Proteins were extracted from thawed spermatozoa, as described previously [26]. Briefly, after washing the pellet containing spermatozoa with PBS, the pellet was sonicated in 300 μL of lysis buffer (2 M thiourea, 7 M urea and 4% 3-[(3-cholamidopropyl)-dimethylammonio]-1-propanesulfonate [CHAPS] and 0.5% a protease inhibitor cocktail) for protein extraction. Aliquots of spermatozoa extract and supernatant of thawed semen containing approximately 700 µg were subjected to a clean-up procedure with a Clean-Up kit (GE Healthcare, Uppsala, Sweden). The protein concentration before and after precipitation was determined by using a Coomassie Plus Kit (Thermo Fisher Scientific, Waltham, MA, USA).

### 2.3. Analysis of Sperm Motility, Viability and Oxidative Stress

Spermatozoa motility parameters were measured by computer-aided sperm analysis (CASA, Hobson Vision Ltd., Baslow, UK) [30]. Video recordings were made using a microscope with a 10× negative phase lens and a Sony CCD black-and-white video camera at a 50 Hz frame rate. For CASA analysis, 3 µL of thawed semen at a concentration of 48 ± 4 × 10^6^ spermatozoa/mL was immediately loaded on Leja four-chamber slides (20 µm deep, IMV Technologies) mounted on a heated stage (37 °C). The value for each sample represents a mean from two separate measurements of motility parameters of 50 spermatozoa. The sperm concentration of thawed semen was measured by flow cytometry. The sperm motility parameters were as follows: percentage of motile sperm (%; MOT), straight-line velocity (μm/s; VSL), curvilinear velocity (μm/s; VCL), LIN-linearity (%, 100 VSL/VCL), average path velocity (μm/s; VAP), the amplitude of lateral head displacement (μm; ALH). The viability, number of spermatozoa and oxidative stress (ROS-positive cells) of cryopreserved sperm were measured using a Muse Cell Analyser (Millipore, Billerica, MA, USA) [31].

### 2.4. 2D-DIGE and Mass Spectrometry Analysis

2D-DIGE analyses were performed to compare (i) the protein profiles of thawed spermatozoa from young and adult bulls (*n* = 6) and (ii) the protein profiles of supernatant of thawed bull semen from young and adult bulls (*n* = 6). 2D-DIGE details were described previously [26,31]. The mixing scheme of samples is presented in Supplementary Table S1. To properly cut the spot of interest, DIGE gels were restrained using Coomassie Brilliant Blue G-250 (Bio-Rad, Hercules, CA, USA). Then the gels were washed with water, and protein spots were manually cut from gels. The gel pieces were subjected to trypsin digestion as described by Westfalewicz et al. [26]. Briefly, spots were de-stained with 50% acetonitrile in 50 mM ammonium bicarbonate, dried, and 2 µL of 0.2 µg/mL trypsin (Promega, Madison, WI, USA) solution were added and incubated overnight at 37 °C. After digestion, peptides were extracted with 0.1% trifluoroacetic acid and desalted with Zip-Tip C18 pipette tips. Peptide samples were analyzed with matrix-assisted laser desorption/ionization time-of-flight tandem (MALDI-TOF/TOF) mass spectrometer (Autoflex Speed, Bruker Daltonics) [15]. The MS and MS/MS spectra from each spot were searched against the National Centre for Biotechnology Information using a Mascot Server (Matrix Science, London, UK) with the following criteria: cleavage enzyme: trypsin; peptide mass tolerance: 100 ppm; fragment ion mass tolerance: 0.5 Da; max missed cleavages: 2; oxidation of methionine as a variable modification and alkylation of cysteine by carbamidomethylation as a fixed modification. The search results were filtered with a significant threshold of *p* < 0.05 and a Mascot ion score cut-off of ≥30 for at least two peptides.

### 2.5. Statistical Analysis

Statistical analyses of motility characteristics and oxidation were compared using paired t-test with Statistica software (StatSoft Inc., Tulsa, OK, USA); the significance level was set at 0.05. Data percentages were transformed by arcsine square-root transformations. All analyses were performed on six biological replicates and two technical replicates besides the 2D-DIGE experiment.

Statistical analysis of changes in protein abundance was performed using the Biological Variance Module of DeCyder Differential In-Gel Analysis version 5.02 software (GE Healthcare) on six biological replicates (individual bulls). Data are expressed as the log standardized abundance to ensure a normal distribution of the data. Paired Student’s *t*-test and the average ratio test were performed; changes in protein spot abundance were considered statistically significant at *p* < 0.05.

Functional associations of differentially abundant proteins were performed using Ingenuity pathway analysis (IPA; Qiagen Silicon Valley, Redwood City, CA, USA) and Search Tool for the Retrieval of Interacting Genes/Proteins (STRING, access date 4 November 2020) (http://string-db.org) [32]. IPA used the core analysis mode to interpret biological functions and pathways. The significance of biological functions and canonical pathways was tested automatically by the software using Fisher’s exact test.

## 3. Results

### 3.1. Bull Semen Parameters

All bulls were fertile, as ensured by the Breeding and Insemination Station. No significant changes in the quality of semen between young and adult bulls were recorded, with the exception of average path velocity (*p* = 0.034), which was higher in adult bulls. Full CASA parameters and flow cytometry results are presented in Table 1 and Supplementary Figure S2.

### 3.2. Cryopreserved Semen Proteome Changes between Young and Adult Bulls

**Figure 1:**
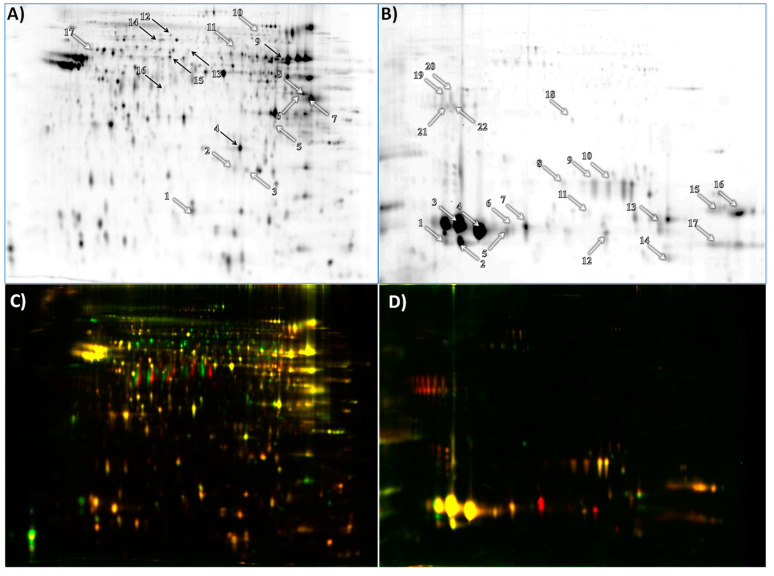
A representative 2D-DIGE gel showing the profile of bull thawed spermatozoa proteome (**A**) and supernatant of thawed sperm proteome (**B**). The overlay of spermatozoa (**C**) and supernatant (**D**) of semen collected from young and mature males. Mature samples were labeled with Cy5 (red) and young samples with Cy3 (green) dyes. White arrows mark protein spots increasing in abundance in adult bulls in comparison to young bulls, and black arrows mark protein spots decreasing in abundance in adult bulls in comparison to young bulls. Arrow numbers correspond to protein spot numbers presented in Table 2 and Table 3.

Analysis of 2D-DIGE of thawed spermatozoa of young and adult bulls showed significant changes in the abundance of 25 protein spots. Among them, 17 were successfully identified. Ten protein spots, representing nine proteins, were more abundant in adult bulls. Zona pellucida binding protein 1 (ZBP1) was represented by two proteoforms. Seven protein spots, representing six proteins, were more abundant in young bulls (Figure 1A, Table 2). Tubulin beta 2B chain (TBB2B) was present in two proteoforms.

A comparison of the supernatant of cryopreserved semen from young and adult bulls showed significant changes in the abundance of 21 protein spots, representing 10 proteins. All of the identified proteins increased in abundance in the supernatant of cryopreserved semen from adult bulls (Figure 1B, Table 3). Metalloproteinase inhibitor 2 (TIMP2), seminal ribonuclease (SRN) and spermadhesin 1 (SPADH1) were present in two proteoforms; binder of sperm protein 1 (BSP1) was present in three; spermadhesin Z13 precursor (SPADH2) and clusterin preproprotein (CLU) were present in four proteoforms.

### 3.3. Functional Analysis of Differentially Abundant Semen Proteins of Young and Adult Bulls

IPA allowed for the detection of molecular and cellular functions associated with proteins of both thawed spermatozoa and supernatant that differentiated young and adult bulls. The top functions selected by the software are presented in Table 4. Full information on molecular and cellular functions is presented in Supplementary Tables S2 and S3.

**Figure 2:**
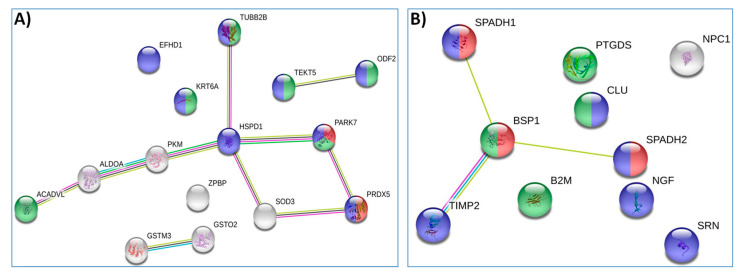
STRING analysis of identified bull thawed spermatozoa proteins (**A**) and supernatant (**B**). The proteins are connected with confidence network edges, indicating putative protein interactions.

STRING neighborhood analysis showed that, among thawed spermatozoa proteins differentiating young and adult bulls, eight could be connected to an evidence-based network (Figure 2A). Two of the networked proteins, protein/nucleic acid deglycase DJ-1 (PARK7) and peroxiredoxin-5 mitochondrial (PRDX5), were indicated as involved in peroxiredoxin activity. Analysis of the supernatant of thawed semen proteins differentiating young and adult bulls showed that four proteins could be connected to an evidence-based network (Figure 2B). Three of the networked proteins, BSP1, SPADH1 and SPADH2, were indicated as involved in the single fertilization biological process.

**Figure 3:**
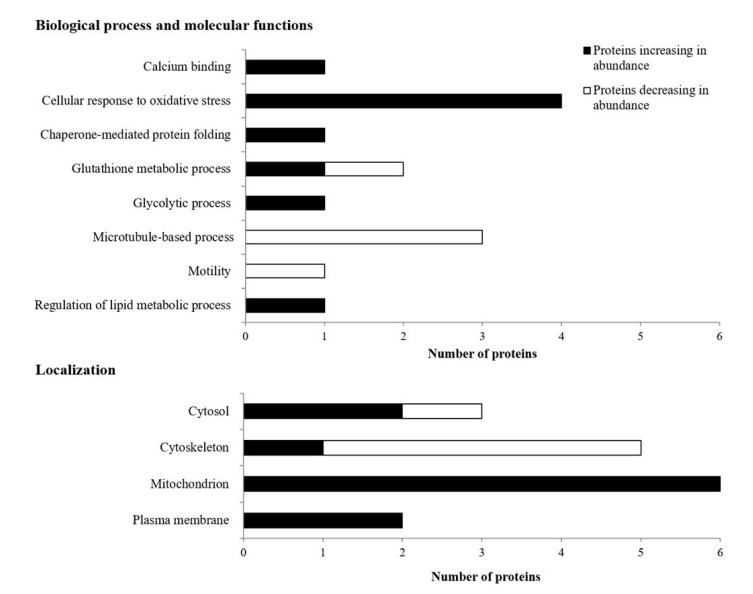
A graph representing number of identified thawed bull spermatozoa proteins from young involved in different biological processes and molecular functions, as well as their cellular location. Black bars represent proteins increasing in abundance in adult bulls in comparison to young bulls, and white bars represent proteins decreasing in abundance in adult bulls in comparison to young bulls.

On the basis of IPA and STRING analyses, as well as data available both in the Uniprot database (http://www.uniprot.org, accessed date 4 November 2020) and in the existing literature, we categorized the identified cryopreserved semen proteins based on their biological process, metabolic function and, in case of spermatozoa proteins, cellular localization (Figure 3). In thawed spermatozoa, most of the proteins that increased in abundance in adult bulls were involved in the response to oxidative stress (4 proteins: glutathione S-transferase omega 2 (GSTO2); PRDX5; PARK7; superoxide dismutase (SODC)). On the other hand, proteins that decreased in abundance in adult bulls were mostly involved in processes connected to the functionality of microtubules (3 proteins: keratin, type II cytoskeletal 59 kDa, component IV (K2C4); outer dense fiber protein 2 (ODF2); tektin-5 (TEKT5); TBB2B) (Figure 3). Proteins from thawed bull spermatozoa could be characterized as intracellular, with the majority of the proteins that increased in abundance in adult bulls being mitochondrial proteins and the majority of the proteins that decreased in abundance localized in the cytoskeleton.

**Figure 4:**
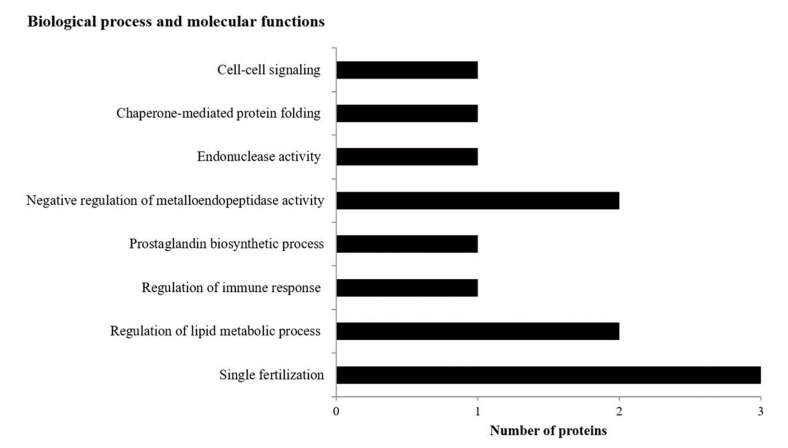
A graph representing number of identified proteins in supernatant from young and adult bulls involved in different biological processes and molecular functions. All of the proteins were increasing in abundance in adult bulls in comparison to young bulls.

In the supernatant of thawed sperm, most proteins that increased in abundance in adult bulls were involved in fertilization (3 proteins: BSP1; SPADH1; SPADH2), followed by the negative regulation of metalloendopeptidase activity (2 proteins: TIMP2; beta-nerve growth factor (NGF)) and the regulation of lipid metabolism (2 proteins: CLU; NPC intracellular cholesterol transporter 2 (NPC2)) (Figure 4). Supernatant proteins could be categorized as either extracellular or sperm surface proteins.

## 4. Discussion

In this study, we have shown the difference in the proteome of young and adult cryopreserved bull semen. In thawed spermatozoa, we identified 15 proteins that differentiate young and adult bulls, of which nine were more abundant in adult bulls, and six were more abundant in young bulls. In the case of the supernatant of thawed sperm, ten proteins that differentiate young and adult bulls were identified, with all of them more abundant in adult bull semen.

Our results showed no significant differences between the number of spermatozoa undergoing oxidative stress, the number of viable spermatozoa and the spermatozoa motility parameters in young and adult bull thawed semen, with the exception of average path velocity. The current knowledge concerning age-related changes in overall bull sperm quality is limited. Studies have shown no effect of age on the motility and oxidation of spermatozoa of Simmental and Nellore bulls [11,33], which was confirmed in our study, with lowered motility and oxidation of adult Nellore bull spermatozoa [8] and higher motility in the spermatozoa of adult Swedish Red and White bulls [6]. This appears to show that viability, oxidation status and motility parameters are not reliable indicators of an age effect in thawed bull sperm.

Despite the similarities in motility parameters and oxidation levels, the protein changes identified in our study suggest that the maturation process of spermatozoa at the biochemical level did progress with age. The proteins of the supernatant of thawed semen increased in abundance with age, and all were either secreted or sperm surface proteins, which strongly suggests that observed changes of thawed supernatant of sperm proteome reflect the changes occurring in the seminal plasma proteome. Among the proteins increasing in abundance, there were major seminal plasma proteins [13,14,15], most of which could be connected to important reproductive and/or physiological roles in bull semen [34]. BSP1 is the most abundant bovine seminal plasma protein, produced by seminal vesicles and secreted to the seminal plasma, where they bind with sperm [35,36]. BSP1 seems to be crucial for the fertility and cryopreservation of bull semen. BSP1, CLU, NGF, SPADH1, SPADH2, SRN and TIMP2 are all proteins capable of binding to mammalian spermatozoa cell membranes influencing their functionality [37,38,39,40,41,42,43] and were identified as bull sperm surface proteins [44,45]. The latter has been recently identified as significant in genome-wide association studies for age at puberty in cattle [46]. It should be emphasized that all of those proteins also indicated a decreased abundance in spermatozoa due to cryopreservation in our previous study, which connects the reduction of their abundance in semen with reduced spermatozoa quality [26]. PTGDS lipophilic properties have been connected to increased fertility of bulls [13], with NPC2 also potentially influencing semen lipid metabolism [47]. Beta 2 macroglobulin was connected to increased sperm count [48]. In conclusion, compared to the thawed semen of the young bulls, the thawed semen of adult bulls is better equipped with major seminal plasma proteins, which could serve as surface proteins for spermatozoa.

The majority of the proteins of thawed spermatozoa differentiating young and adult bulls increased in abundance with age, and all of them can be related to the higher maturity of spermatozoa cells. GSTO2, PARK7, PRDX5 and SODC were attributed to being involved in the oxidative stress reaction of spermatozoa. The control of semen oxidation levels is crucial for fertilization; while low levels of reactive oxygen species (ROS) positively influence sperm activation, high levels of ROS can lead to infertility [49], especially after cryopreservation, which negatively impacts the activity of antioxidant enzymes [50]. An increase in antioxidative proteins in the spermatozoa of adult bulls suggests that antioxidative protection is well-developed compared to the semen of younger bulls. This increase can also participate in the antioxidative defense of spermatozoa during the process of cryopreservation. Zona pellucida-binding protein 1, named for its function, is involved in the process of acrosome membrane binding to an oocyte and has been connected with correct sperm head morphology [51]. 60 kDa heat shock protein, a molecular chaperone produced in response to a variety of stresses, could possibly be involved in modulating the immunological response of the female reproductive tract [52]. Fructose-bisphosphate aldolase A and pyruvate kinase are both enzymes involved in glycolysis, and therefore, they influence energy production in the spermatozoa cell [53]. In summary, spermatozoa proteins that increase in abundance in adult bulls can be connected to energy production, the protection of spermatozoa in both male and female reproductive tracts, protection against oxidative stress and fertilizing ability. This indicates their higher maturity compared to the spermatozoa of young bulls and might contribute to an increased ability to fertilize an oocyte.

All of the spermatozoa proteins that dropped in abundance with age and were thus more abundant in young bull spermatozoa, with the exception of GSTM3, were connected to the cytoskeleton and its developmental processes. ODF2 and TBB2B are the constituents of microtubules that take part in constructing the axoneme of spermatozoa [54,55]. K2C4 takes part in the creation of the motile cytoskeleton of epithelia [56], and it has been shown that a keratin-like protein of similar molecular weight as K2C4 is present during meiotic and post-meiotic stages of spermatogenesis [57]. Tektin 5 was suggested as a protein important both for the formation of the flagellum as well as motility [58]. EFHC1, a microtubule-associated protein, is involved in the cell division process [59]. The larger abundance of those proteins in the spermatozoa of young bulls strongly suggests that developmental processes are still in progress and that the building of inner structures of the cells, especially axoneme, is not completed in the spermatozoa of younger bulls.

The importance of having a higher abundance of GSTM3 in the spermatozoa of younger bulls is unknown; however, the inhibition of the Mu class of glutathione S-transferases (GST) on goat spermatozoa has led to lowered fertilization rates after sperm capacitation [60]. It is interesting to note that in adult bull spermatozoa, different classes of GST and GSTO2 were identified as increasing in abundance. This suggests that the maturation of spermatozoa can also be related to a rearrangement in the abundance of enzymes from particular protein groups. Further studies are necessary in order to fully understand the importance of an abundance change in GST proteins between young and adult bull spermatozoa.

## 5. Conclusions

In conclusion, our results have shown the change in proteome composition between thawed sperm of young and adult bulls. Cryopreserved adult bull semen seems to be better equipped with major seminal plasma proteins, which serve as surface proteins for spermatozoa. Adult bull spermatozoa proteins seem to be better equipped with proteins connected to energy production, the protection of spermatozoa in both male and female reproductive tracts, protection against oxidative stress and fertilizing ability, which indicate their higher maturity. On the other hand, proteins increasing in abundance in young bull spermatozoa were connected to the cytoskeleton and its development, which strongly suggests that developmental processes are still in progress.

## Figures and Tables

**Table 1:** Viability, reactive oxygen species generation and CASA parameters (mean ± SD) of thawed bull semen in relation to their age; VCL = curvilinear velocity; VSL = straight line velocity; VAP = average path velocity; LIN = linearity; ALH = amplitude of lateral head displacement; *n* = 6 in each group. Different letters indicate significant differences between groups (*p* ≤ 0.05).

Parameters	Young	Adult
Viability (%)	37.85 ± 11.45	39.79 ± 8.61
Oxidation (%)	72.86 ± 12.84	71.58 ± 10.88
Motility (%)	43.79 ± 12.52	38.33 ± 6.47
VCL (μm/s)	89.17 ± 4.99	105.53 ± 17.31
VAP (μm/s)	51.86 ^a^ ± 5.65	62.41 ^b^ ± 11.53
VSL (μm/s)	22.02 ± 3.16	30.87 ± 13.82
LIN (%)	17.53 ± 1.60	22.30 ± 7.36
ALH (μm)	1.80 ± 0.67	2.66 ± 1.49

**Table 2:** Proteins changing in thawed spermatozoa of adult bulls in comparison to young bulls.

Protein Spot No.	Protein Name	Gene Name	MW ^1^	Calc. pI ^2^	Protein Score	Sequence Coverage (%) ^3^	Ident. Peptides ^4^	*p*-Value	FC ^5^ Adult/Young
1	Peroxiredoxin-5, mitochondrial	*PRDX5*	17,522	5.92	524	72	6	0.0280	1.11
2	Protein/nucleic acid deglycase DJ-1	*PARK7*	20,194	6.84	80	33	2	0.0250	1.18
3	Superoxide dismutase [Cu-Zn]	*SODC*	24,794	8.70	120	31	2	0.0200	1.12
4	Glutathione S-transferase, mu 3	*GSTM3*	27,174	6.83	766	82	8	0.0310	−1.42
5	Glutathione S-transferase omega 2	*GSTO2*	28,869	7.49	269	43	3	0.0043	1.14
6	Fructose-bisphosphate aldolase A	*ALDOA*	39,925	8.45	457	44	4	0.0260	1.10
7	Zona pellucida-binding protein 1	*ZPBP1*	37,559	9.28	365	28	5	0.0220	1.17
8	Zona pellucida-binding protein 1	*ZPBP1*	37,559	9.28	313	50	3	0.0330	1.14
9	Tektin-5	*TEKT5*	56,302	7.74	214	47	2	0.0250	−1.17
10	Pyruvate kinase PKM	*PKM*	58,482	7.96	210	27	2	0.0300	1.21
11	Very long-chain specific acyl-CoA dehydrogenase. mitochondrial	*ACADVL*	71,003	8.74	204	26	2	0.0150	1.27
12	EF-hand domain-containing protein 1	*EFHC1*	74,441	5.78	312	41	4	0.0120	−1.22
13	Tubulin beta-2B chain	*TBB2B*	50,377	4.78	360	27	4	0.0052	−1.46
14	Tubulin beta-2B chain	*TBB2B*	50,377	4.78	213	28	2	0.0078	−1.38
15	Outer dense fiber protein 2	*ODF2*	76,249	7.52	513	37	5	0.0230	−1.10
16	Keratin, type II cytoskeletal 59 kDa, component IV	*K2C4*	61,275	8.58	91	8	2	0.0060	−1.16
17	60 kDa heat shock protein, mitochondrial	*HSPD1*	61,110	5.71	317	39	3	0.0310	1.16

^1^ MW = predicted molecular weight. ^2^ Calc. pI = isoelectric point calculated on the basis of amino-acid sequence. ^3^ Sequence coverage = percentage of the amino-acid sequence identified. ^4^ Ident. peptides = number of peptides identified with *p* < 0.05. ^5^ FC = fold change, a positive ratio denotes proteins more abundant while a negative ratio denotes proteins less in adult bull spermatozoa.

**Table 3:** Proteins changing in supernatant of thawed sperm of adult bulls in comparison to young bulls.

Protein Spot No.	Protein Name	Gene Name	MW ^1^	Calc. pI ^2^	Protein Score	Sequence Coverage (%) ^3^	Ident. Peptides ^4^	*p*-Value	FC ^5^ Adult/Young
1	Spermadhesin-1	*SPADH1*	13,141	5.04	540	97	5	0.0180	1.41
2	Spermadhesin-1	*SPADH1*	13,141	5.04	479	71	4	0.0280	1.36
3	Binder of sperm protein 1	*BSP1*	13,244	5.08	207	53	1	0.0410	1.30
4	Binder of sperm protein 1	*BSP1*	13,244	5.08	379	53	2	0.0340	1.30
5	Binder of sperm protein 1	*BSP1*	13,244	5.08	145	22	1	0.0120	1.42
6	spermadhesin Z13 precursor	*SPADH2*	15,496	5.92	386	52	3	0.0110	1.29
7	spermadhesin Z13 precursor	*SPADH2*	15,496	5.92	307	52	2	0.0052	1.42
8	Prostaglandin-H2 D-isomerase	*PTGDS*	21,444	6.43	120	21	2	0.0037	1.54
9	metalloproteinase inhibitor 2	*TIMP2*	25,112	7.44	241	48	2	0.0077	1.42
10	metalloproteinase inhibitor 2	*TIMP2*	25,112	7.44	387	63	3	0.0150	1.37
11	spermadhesin Z13 precursor	*SPADH2*	15,496	5.92	266	42	2	0.0160	1.24
12	spermadhesin Z13 precursor	*SPADH2*	15,496	5.92	343	42	3	0.0027	1.41
13	NPC intracellular cholesterol transporter 2	*NPC2*	14,948	7.81	296	60	2	0.0290	1.25
14	Beta-2-microglobulin	*B2M*	11,151	7.08	137	81	2	0.0016	1.53
15	Seminal ribonuclease	*SRN*	14,173	9.04	95	61	1	0.0210	1.53
16	Seminal ribonuclease	*SRN*	14173	9.04	174	64	2	0.0170	1.61
17	Beta-nerve growth factor	*NGF*	26,995	9.72	298	51	4	0.0360	1.29
18	clusterin preproprotein	*CLU*	51,651	5.73	244	30	2	0.0320	1.37
19	clusterin preproprotein	*CLU*	51,651	5.73	306	26	3	0.0430	1.40
20	clusterin preproprotein	*CLU*	51,651	5.73	233	23	2	0.0250	1.49
21	clusterin preproprotein	*CLU*	51,651	5.73	243	23	2	0.0410	1.43

^1^ MW = predicted molecular weight. ^2^ Calc. pI = isoelectric point calculated on the basis of amino-acid sequence. ^3^ Sequence coverage = percentage of the amino-acid sequence identified. ^4^ Ident. peptides = number of peptides identified with *p* < 0.05. ^5^ FC = fold change, a positive ratio denotes proteins more abundant while a negative ratio denotes proteins less in adult bull supernatant.

**Table 4:** Top molecular and cellular functions performed by proteins changing in adult bulls thawed spermatozoa and supernatant of thawed semen in comparison to young bulls thawed spermatozoa and supernatant of thawed semen.

Function Name	*p*-Value Range	Number of Proteins
*Thawed spermatozoa*		
Energy Production	3.23 × 10^−2^–6.99 × 10^−7^	5
Nucleic Acid Metabolism	3.23 × 10^−2^–6.99 × 10^−7^	5
Small Molecule Biochemistry	4.81 × 10^−2^–6.99 × 10^−7^	9
Free Radical Scavenging	4.55 × 10^−2^–1.39 × 10^−6^	3
Molecular Transport	4.81 × 10^−2^–1.39 × 10^−6^	6
*Supernatant of* *thawed semen*		
Cell Morphology	1.28 × 10^−2^–1.44 × 10^−5^	5
Cellular Movement	1.47 × 10^−2^–1.57 × 10^−5^	3
Cellular Development	1.47 × 10^−2^–1.57 × 10^−5^	4
Cellular Function and Maintenance	8.93 × 10^−2^–1.57 × 10^−5^	5
Cellular growth and proliferation	1.47 × 10^−2^–1.57 × 10^−5^	5

## Data Availability

None of the data were deposited in an official repository. Proteomic raw data are available upon request from the corresponding author.

## Supplementary Materials

The following are available online at https://www.mdpi.com/article/10.3390/ani11072013/s1, Figure S1: Programmed and recorded freezing curve of bull semen; dotted line–programmed rate of freezing, continuous line–recorded rate of freezing, Figure S2: Representative picture of viability measurement of thawed bull semen, showing three populations of spermatozoa–live, dead, and third intermediate population, marked with blue oval, Table S1: Mixing and dying scheme of samples of spermatozoa extract and supernatant of thawed bull sperm from young and adult bulls; *n* = 6 for each group, Table S2: Molecular and cellular functions performed by proteins changing in adult bulls thawed spermatozoa in comparison to young bulls thawed spermatozoa, Table S3: Molecular and cellular functions performed by proteins changing in adult bulls supernatant of thawed semen in comparison to young bulls supernatant of thawed semen.
