# Supplementary material for: Comparative Proteomic Analysis of Young and Adult Bull (Bos taurus) Cryopreserved Semen
Source: Animals (Basel). 2021 Jul 5;11(7):2013. doi: 10.3390/ani11072013 (PMC8300238; doi:10.3390/ani11072013)
Supplement: Supplementary file 1 [file animals-11-02013-s001.zip › animals-1249657-supplementary.pdf]

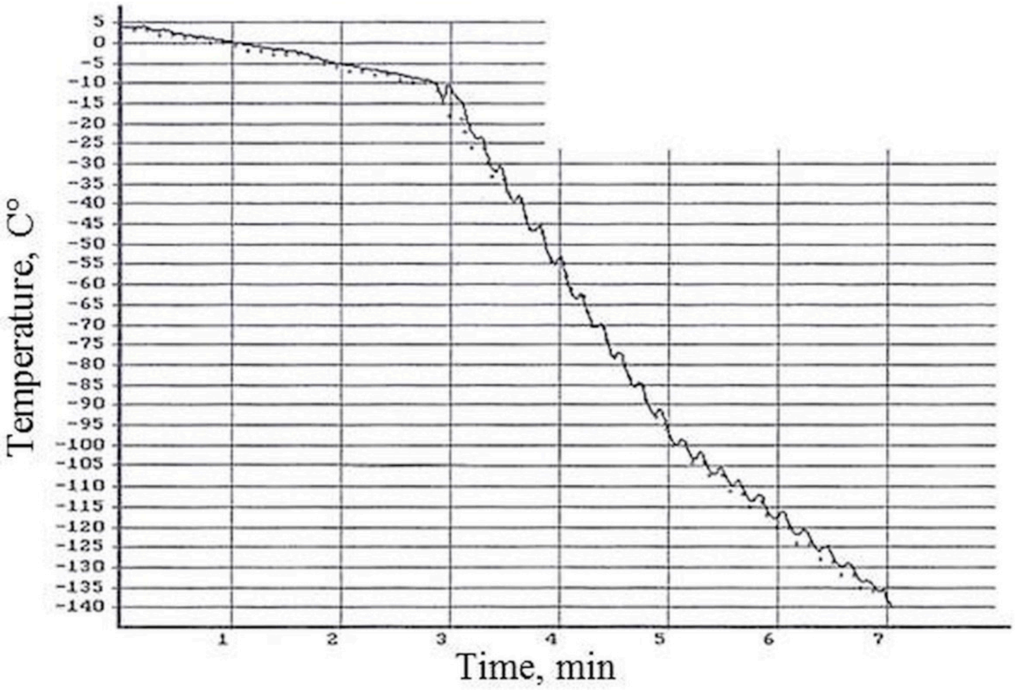

Fig. S1. Programmed and recorded freezing curve of bull semen; dotted line – programmed rate of freezing, continuous line – recorded rate of freezing.

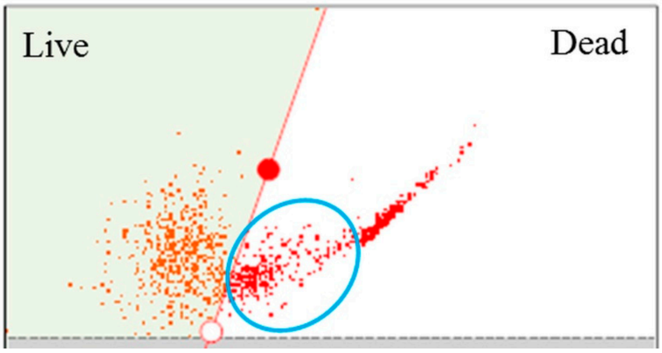

Fig. S2. Representative picture of viability measurement of thawed bull semen, showing three populations of spermatozoa – live, dead, and third intermediate population, marked with blue oval.

Table S1. Mixing and dying scheme of samples of spermatozoa extract and supernatant of thawed bull sperm from young and adult bulls; N = 6 for each group

Gel #	Cy2	Cy3	Cy5
1	Pooled Standard	Young 1	Adult 3
2	Pooled Standard	Adult 1	Young 4
3	Pooled Standard	Young 2	Adult 4
4	Pooled Standard	Adult 2	Young 5
5	Pooled Standard	Young 3	Adult 6
6	Pooled Standard	Adult 5	Young 6

Table S2. Molecular and cellular functions performed by proteins changing in adult bulls cryopreserved spermatozoa in comparison to young bulls cryopreserved spermatozoa

Categories	Diseases or Functions Annotation	p-Value	Molecules	# Molecules
Energy Production, Nucleic Acid Metabolism, Small Molecule Biochemistry	Synthesis of ATP	6.99E-07	ALDOA, HSPD1, PKM, SOD1	4
Free Radical Scavenging, Molecular Transport	Quantity of hydrogen peroxide	1.39E-06	PARK7, PRDX5, SOD1	3
Free Radical Scavenging, Molecular Transport	Accumulation of hydrogen peroxide	1.22E-05	PRDX5, SOD1	2
Free Radical Scavenging	Modification of hydrogen peroxide	3.39E-05	PRDX5, SOD1	2
Free Radical Scavenging, Small Molecule Biochemistry	Metabolism of hydrogen peroxide	9.39E-05	PARK7, PRDX5, SOD1	3
Cellular Function and Maintenance	Homeostasis of muscle cells	9.99E-05	ALDOA, SOD1	2
Lipid Metabolism, Small Molecule Biochemistry	Synthesis of fatty acid	1.38E-04	ACADVL, PARK7, PKM, SOD1	4
Free Radical Scavenging, Small Molecule Biochemistry	Degradation of hydrogen peroxide	1.40E-04	PRDX5, SOD1	2
Carbohydrate Metabolism, Energy Production	Metabolism of pyruvic acid	1.75E-04	ALDOA, PKM	2
Cellular Function and Maintenance	Respiration of mitochondria	3.35E-04	PKM, SOD1	2
Cell Morphology, Cellular Function and Maintenance	Mitochondrial membrane potential	3.70E-04	PARK7, SOD1	2
Cellular Assembly and Organization	Binding of zona pellucida	3.87E-04	ALDOA, ZPBP2	2
Drug Metabolism, Protein Synthesis	Metabolism of glutathione	4.63E-04	GSTM3, SOD1	2
Cell-To-Cell Signaling and Interaction, Reproductive System Development and Function	Binding of sperm	5.67E-04	ALDOA, ZPBP2	2
Cell Morphology, Cellular Function and Maintenance	Transmembrane potential of mitochondria	7.43E-04	HSPD1, PARK7, SOD1	3
Drug Metabolism	Metabolism of xenobiotic	1.05E-03	GSTM3, GSTO2	2
Amino Acid Metabolism, Small Molecule	Synthesis of amino acids	1.27E-03	PARK7, PKM	2

Biochemistry				
Organismal Injury and Abnormalities	Oxidative stress	1.51E-03	PRDX5, SOD1	2
Cell Death and Survival	Cell death of nervous tissue cell lines	1.65E-03	PARK7, SOD1	2
Cell Signaling, Small Molecule Biochemistry	Synthesis of nitric oxide	1.69E-03	HSPD1, PARK7, SOD1	3
Drug Metabolism, Molecular Transport, Small Molecule Biochemistry	Concentration of glutathione	1.96E-03	PKM, SOD1	2
Cell Death and Survival, Organismal Injury and Abnormalities	Cell death of epithelial cell lines	1.96E-03	HSPD1, PARK7, SOD1	3
Carbohydrate Metabolism, Small Molecule Biochemistry	Metabolism of D-hexose	2.24E-03	ALDOA, PKM	2
Cell Death and Survival	Cell viability of brain cells	2.60E-03	PARK7, SOD1	2
Cell Morphology, Cellular Assembly and Organization	Abnormal morphology of Nucleus	2.64E-03	SOD1, ZPBP2	2
Cell-To-Cell Signaling and Interaction, Drug Metabolism, Molecular Transport, Small Molecule Biochemistry	Concentration of dopamine	2.92E-03	PARK7, SOD1	2
Cellular Assembly and Organization, Cellular Function and Maintenance	Organization of cytoplasm	3.35E-03	ALDOA, EFHD1, KRT6A, PARK7, PKM, SOD1	6
Behavior	Locomotion	3.41E-03	ACADVL, PARK7, SOD1	3
Cell Signaling	Activation of enzyme	3.75E-03	HSPD1, PARK7, SOD1	3
Carbohydrate Metabolism	Glycolysis of cells	4.66E-03	ALDOA, PKM	2
Energy Production, Molecular Transport, Nucleic Acid Metabolism, Small Molecule Biochemistry	Concentration of ATP	4.84E-03	ALDOA, PKM	2
Cell Morphology, Cellular Assembly and Organization	Morphology of mitochondria	5.15E-03	HSPD1, SOD1	2
Organismal Functions	Energy expenditure	5.53E-03	ACADVL, SOD1	2
Carbohydrate Metabolism	Accumulation of carbohydrate	5.93E-03	PKM, SOD1	2
Embryonic Development, Organismal Development, Reproductive System Development and Function	Fertilization	6.62E-03	PARK7, ZPBP2	2
Inflammatory Response	Inflammatory response	6.82E-03	HSPD1, PARK7, PRDX5, SOD1	4
Cell Death and Survival	Apoptosis of neurons	7.24E-03	HSPD1, PARK7, SOD1	3
Cell Death and Survival	Cell viability of carcinoma cell lines	7.80E-03	PARK7, PKM	2

Cellular Compromise, Cellular Function and Maintenance	Endoplasmic reticulum stress response	8.10E-03	HSPD1, SOD1	2
Inflammatory Response, Organismal Injury and Abnormalities	Inflammation of organ	8.26E-03	ACADVL, ALDOA, KRT6A, PKM, SOD1	5
Cell Death and Survival	Cell death of hepatoma cell lines	9.72E-03	PKM, SOD1	2
Organ Morphology, Skeletal and Muscular System Development and Function	Morphology of skeletal muscle	1.08E-02	ACADVL, SOD1	2
Cellular Movement	Migration of cells	1.08E-02	ALDOA, HSPD1, PARK7, PKM, SOD1, TUBB2B	6
Lipid Metabolism, Small Molecule Biochemistry	Homeostasis of lipid	1.08E-02	ACADVL, SOD1	2
Protein Degradation, Protein Synthesis	Stabilization of protein	1.10E-02	HSPD1, PARK7	2
Cell Death and Survival	Cell death of neuroblastoma cell lines	1.11E-02	PARK7, SOD1	2
Skeletal and Muscular System Development and Function	Morphology of muscle	1.19E-02	ACADVL, PARK7, SOD1	3
Cellular Assembly and Organization, Cellular Function and Maintenance	Organization of cytoskeleton	1.20E-02	ALDOA, EFHD1, KRT6A, PKM, SOD1	5
Carbohydrate Metabolism	Quantity of carbohydrate	1.32E-02	ACADVL, PKM, SOD1	3
DNA Replication, Recombination, and Repair	DNA damage	1.41E-02	PARK7, SOD1	2
Cellular Assembly and Organization, Cellular Function and Maintenance	Organization of filaments	1.47E-02	ALDOA, SOD1	2
Cellular Assembly and Organization	Organization of organelle	1.61E-02	ALDOA, PARK7, SOD1	3
Cell Death and Survival	Cell death of connective tissue cells	1.73E-02	HSPD1, PARK7, SOD1	3
Cell-To-Cell Signaling and Interaction, Hematological System Development and Function, Immune Cell Trafficking, Inflammatory Response	Activation of macrophages	1.77E-02	HSPD1, SOD1	2
Cell Death and Survival, Nervous System Development and Function	Cell viability of neurons	1.77E-02	PARK7, SOD1	2
Skeletal and Muscular System Development and Function	Muscle contraction	1.84E-02	ALDOA, SOD1	2
Protein Degradation, Protein Synthesis	Catabolism of protein	1.92E-02	HSPD1, PARK7, SOD1	3
Cellular Function and Maintenance	Cellular homeostasis	1.92E-02	ALDOA, HSPD1, PARK7, PKM, SOD1	5
Carbohydrate Metabolism	Metabolism of carbohydrate	1.94E-02	ALDOA, GSTO2, PKM	3
Cell Signaling, Post-Translational Modification	Activation of Protein	1.98E-02	PARK7, SOD1	2

	kinase			
Cell Death and Survival	Cytolysis	2.19E-02	ALDOA, KRT6A	2
Cell Morphology, Organismal Injury and Abnormalities	Hypertrophy of cells	2.59E-02	PARK7, SOD1	2
Cell Morphology, Cellular Function and Maintenance	Autophagy of cells	2.77E-02	PKM, SOD1	2
Cell Death and Survival	Apoptosis of tumor cell lines	3.19E-02	HSPD1, PARK7, PKM, SOD1	4
Cellular Growth and Proliferation	Colony formation of tumor cell lines	3.21E-02	PKM, SOD1	2
Nervous System Development and Function	Sensation	3.47E-02	PKM, SOD1	2
Cell Death and Survival	Cell death of carcinoma cell lines	3.68E-02	PKM, SOD1	2
Cell Death and Survival	Apoptosis	4.15E-02	ALDOA, HSPD1, PARK7, PKM, PRDX5, SOD1	6
Reproductive System Development and Function	Fertility	4.17E-02	SOD1, ZPBP2	2
Free Radical Scavenging	Production of reactive oxygen species	4.25E-02	PARK7, SOD1	2
Cell Death and Survival, Embryonic Development	Cell death of embryonic cell lines	4.28E-02	PARK7, SOD1	2
Cell Death and Survival	Cell death	4.37E-02	ALDOA, HSPD1, KRT6A, PARK7, PKM, PRDX5, SOD1	7
Cell Death and Survival	Cell viability	4.82E-02	HSPD1, PARK7, PKM, SOD1	4

Table S3. Molecular and cellular functions performed by proteins changing in adult bulls supernatant of cryopreserved semen in comparison to young bulls supernatant of cryopreserved semen

Categories	Diseases or Functions Annotation	p-Value	Molecules	# Molecules
Cell Morphology	Morphology of leukocytes	2.40E-06	B2M, NGF, NPC2, PTGDS	4
Cell Morphology, Inflammatory Response	Morphology of phagocytes	1.14E-05	NGF, NPC2, PTGDS	3
Cell Morphology, Hematological System Development and Function, Hypersensitivity Response, Inflammatory Response	Morphology of mast cells	1.44E-05	NGF, PTGDS	2
Cardiovascular System Development and Function, Cell Morphology, Cellular Development, Cellular Function and Maintenance, Cellular Growth and Proliferation, Organismal Development, Tissue Development	Morphogenesis of vascular endothelial cells	1.57E-05	NGF, TIMP2	2
Lipid Metabolism, Small Molecule Biochemistry	Synthesis of prostaglandin	1.64E-05	NGF, NPC2, PTGDS	3
Lipid Metabolism, Small Molecule Biochemistry	Synthesis of prostaglandin D2	3.48E-05	NPC2, PTGDS	2
Cell Death and Survival	Apoptosis of oligodendrocytes	5.34E-05	NGF, PTGDS	2
Hematological System Development and Function, Immune Cell Trafficking, Inflammatory Response, Tissue Development	Accumulation of leukocytes	7.18E-05	B2M, PTGDS, TIMP2	3
Molecular Transport, Small Molecule Biochemistry	Release of histamine	7.28E-05	B2M, NGF	2
Cellular Movement, Hematological System Development and Function, Immune Cell Trafficking, Inflammatory Response	Migration of phagocytes	8.16E-05	NGF, PTGDS, TIMP2	3
Immunological Disease	Abnormal morphology of immune system	1.19E-04	B2M, NPC2, PTGDS	3
Hematological System Development and Function, Organismal Functions	Flow of blood	1.21E-04	NGF, PTGDS	2
Cell-To-Cell Signaling and Interaction, Drug Metabolism, Molecular Transport, Small Molecule	Release of dopamine	1.58E-04	NGF, TIMP2	2

Biochemistry				
Cell Death and Survival	Cell death of leukemia cell lines	1.58E-04	B2M, NGF, NPC2	3
Organismal Injury and Abnormalities, Tissue Morphology	Size of lesion	1.84E-04	NGF, PTGDS, TIMP2	3
Hematological System Development and Function, Tissue Morphology	Quantity of leukocytes	1.96E-04	B2M, NGF, NPC2, TIMP2	4
DNA Replication, Recombination, and Repair	Synthesis of DNA	2.77E-04	NGF, PTGDS, TIMP2	3
Cellular Movement, Hematological System Development and Function, Immune Cell Trafficking, Inflammatory Response	Migration of monocytes	3.11E-04	NGF, TIMP2	2
Nervous System Development and Function, Tissue Morphology	Quantity of neurons	3.30E-04	B2M, NGF, NPC2	3
Hematological System Development and Function, Inflammatory Response, Tissue Morphology	Quantity of phagocytes	3.68E-04	B2M, NGF, TIMP2	3
Cardiovascular System Development and Function, Tissue Morphology	Permeability of blood vessel	3.82E-04	NGF, TIMP2	2
Cell Morphology, Nervous System Development and Function, Tissue Morphology	Size of neurons	3.82E-04	B2M, NGF	2
Hematological System Development and Function, Immune Cell Trafficking, Inflammatory Response, Tissue Development	Accumulation of granulocytes	4.68E-04	PTGDS, TIMP2	2
Cellular Movement	Migration of melanoma cell lines	4.91E-04	NGF, TIMP2	2
Hematological System Development and Function, Tissue Morphology	Quantity of myeloid cells	5.50E-04	B2M, NGF, TIMP2	3
Behavior, Nervous System Development and Function	Circadian rhythm	5.71E-04	NGF, PTGDS	2
Lipid Metabolism, Molecular Transport, Small Molecule Biochemistry	Concentration of eicosanoid	5.71E-04	NGF, PTGDS	2
Organismal Injury and Abnormalities, Tissue Morphology	Volume of lesion	6.22E-04	NGF, TIMP2	2
Cell Morphology	Size of cells	6.66E-04	B2M, NGF, PTGDS	3
Neurological Disease, Organismal Injury and Abnormalities	Degeneration of brain	6.93E-04	NGF, NPC2	2
Cancer, Hematological Disease, Immunological Disease, Organismal Injury and Abnormalities	T-cell non-Hodgkin disease	7.27E-04	B2M, BSPH1, TIMP2	3
Cancer, Hematological Disease, Immunological Disease, Organismal Injury and Abnormalities	T-cell leukemia/lymphoma	7.68E-04	B2M, BSPH1	2

Drug Metabolism, Lipid Metabolism, Small Molecule Biochemistry	Synthesis of prostaglandin E2	7.97E-04	NGF, NPC2	2
Cellular Compromise, Hypersensitivity Response, Inflammatory Response	Degranulation of mast cells	8.16E-04	NGF, PTGDS	2
Neurological Disease	Tremor	9.18E-04	NGF, NPC2	2
Cellular Movement	Cell movement of myeloid cells	9.25E-04	NGF, PTGDS, TIMP2	3
Dermatological Diseases and Conditions, Neurological Disease, Organismal Injury and Abnormalities	Hyperesthesia	9.39E-04	NGF, PTGDS	2
Hematological System Development and Function, Immune Cell Trafficking, Inflammatory Response, Tissue Development	Accumulation of phagocytes	9.60E-04	PTGDS, TIMP2	2
Nervous System Development and Function, Tissue Morphology	Quantity of central nervous system cells	9.82E-04	NGF, NPC2	2
Hematological System Development and Function, Immune Cell Trafficking, Inflammatory Response, Tissue Development	Accumulation of mononuclear leukocytes	1.03E-03	B2M, PTGDS	2
Cell Death and Survival	Necrosis	1.08E-03	B2M, NGF, NPC2, PTGDS, TIMP2	5
Metabolic Disease	Amyloidosis	1.14E-03	B2M, NGF, PTGDS	3
Cellular Development, Connective Tissue Development and Function	Differentiation of fibroblast cell lines	1.28E-03	NPC2, PTGDS	2
Cell Morphology, Hematological System Development and Function	Morphology of myeloid cells	1.37E-03	NPC2, PTGDS	2
Cellular Movement	Migration of myeloid cells	1.54E-03	PTGDS, TIMP2	2
Cell Signaling, Molecular Transport, Nucleic Acid Metabolism, Small Molecule Biochemistry	Concentration of cyclic AMP	1.58E-03	NGF, TIMP2	2
Lipid Metabolism, Molecular Transport, Small Molecule Biochemistry	Secretion of lipid	1.61E-03	NGF, NPC2	2
Lipid Metabolism, Molecular Transport, Small Molecule Biochemistry	Concentration of phospholipid	1.87E-03	NGF, NPC2	2
Hematological System Development and Function, Lymphoid Tissue Structure and Development, Tissue Morphology	Quantity of lymphocytes	1.92E-03	B2M, NGF, NPC2	3
Cell Death and Survival, Cellular Compromise	Cytotoxicity of cells	2.14E-03	B2M, NGF	2
Cell Morphology, Cellular Function and Maintenance	Transmembrane potential of mitochondria	2.17E-03	B2M, NGF	2

Cellular Movement	Invasion of carcinoma cell lines	2.62E-03	NGF, TIMP2	2
Nucleic Acid Metabolism, Small Molecule Biochemistry	Metabolism of cyclic nucleotides	2.80E-03	NGF, TIMP2	2
Lipid Metabolism, Molecular Transport, Small Molecule Biochemistry	Concentration of lipid	2.83E-03	NGF, NPC2, PTGDS	3
Cell Death and Survival, Cellular Compromise, Neurological Disease, Tissue Morphology	Degeneration of neurons	2.86E-03	NGF, NPC2	2
Cellular Function and Maintenance, Hematological System Development and Function	Function of T lymphocytes	3.06E-03	B2M, NPC2	2
Nutritional Disease	Weight loss	3.08E-03	NGF, NPC2	2
Cancer, Hematological Disease, Immunological Disease, Organismal Injury and Abnormalities	Multiple myeloma	3.41E-03	B2M, TIMP2	2
Cell-To-Cell Signaling and Interaction, Nervous System Development and Function	Long-term potentiation	3.48E-03	B2M, NGF	2
Cell Cycle	Arrest in G1 phase	3.54E-03	NGF, TIMP2	2
Behavior	Emotional behavior	3.81E-03	B2M, NGF	2
Embryonic Development, Organismal Development, Tissue Development	Growth of embryonic tissue	3.81E-03	NGF, TIMP2	2
Cell Death and Survival	Apoptosis of leukemia cell lines	3.90E-03	B2M, NGF	2
Cell Death and Survival	Apoptosis of breast cancer cell lines	4.53E-03	B2M, NGF	2
Cell-To-Cell Signaling and Interaction	Activation of cells	4.71E-03	B2M, NGF, NPC2	3
Cellular Growth and Proliferation	Expansion of cells	4.88E-03	B2M, NGF	2
Organismal Injury and Abnormalities	Discomfort	4.92E-03	NGF, TIMP2	2
Immunological Disease, Inflammatory Disease, Inflammatory Response, Neurological Disease, Organismal Injury and Abnormalities	Experimental autoimmune encephalomyelitis	5.18E-03	B2M, NGF	2
Cell Signaling, Molecular Transport, Vitamin and Mineral Metabolism	Mobilization of Ca2+	5.70E-03	B2M, NGF	2
Cardiovascular Disease, Organismal Injury and Abnormalities	Infarction	6.46E-03	NGF, PTGDS	2
Cardiovascular System Development and Function	Development of vasculature	6.73E-03	NGF, NPC2, TIMP2	3
Embryonic Development, Organismal Development	Development of body trunk	7.16E-03	B2M, NGF, NPC2	3
Cardiovascular System Development and Function	Morphology of	7.48E-03	NPC2, TIMP2	2

	vasculature			
Cell Morphology, Cellular Development	Branching of cells	8.43E-03	NGF, TIMP2	2
Organismal Survival	Organismal death	8.93E-03	B2M, NGF, NPC2, TIMP2	4
Immunological Disease	Hypersensitive reaction	9.65E-03	NGF, PTGDS	2
Cellular Development, Nervous System Development and Function, Tissue Development	Differentiation of neurons	9.91E-03	NGF, TIMP2	2
Cell Death and Survival	Apoptosis of neurons	1.04E-02	NGF, PTGDS	2
Nervous System Development and Function	Development of Visual system	1.14E-02	NGF, TIMP2	2
Cell Death and Survival	Apoptosis	1.22E-02	B2M, NGF, PTGDS, TIMP2	4
Infectious Diseases	Replication of RNA virus	1.26E-02	B2M, NGF	2
Cellular Development, Cellular Growth and Proliferation	Cell proliferation of breast cancer cell lines	1.28E-02	NGF, TIMP2	2
Molecular Transport	Quantity of metal	1.29E-02	B2M, NGF	2
Cell Cycle	Mitosis	1.36E-02	NGF, TIMP2	2
Cell Morphology, Nervous System Development and Function, Neurological Disease, Tissue Morphology	Abnormal morphology of neurons	1.38E-02	B2M, NGF	2
